# Morph Matters: Aggression Bias in a Polymorphic Sparrow

**DOI:** 10.1371/journal.pone.0048705

**Published:** 2012-10-31

**Authors:** Brent M. Horton, Mark E. Hauber, Donna L. Maney

**Affiliations:** 1 Department of Psychology, Emory University, Atlanta, Georgia, United States of America; 2 Department of Psychology, Hunter College and the Graduate Center of the City University of New York, New York, New York, United States of America; Arizona State University, United States of America

## Abstract

In species with discrete morphs exhibiting alternative behavioral strategies, individuals may vary their aggressive behavior in competitive encounters according to the phenotype of their opponent. Such aggression bias has been documented in multiple polymorphic species evolving under negative frequency-dependent selection, but it has not been well-studied under other selection regimes. We investigated this phenomenon in white-throated sparrows (*Zonotrichia albicollis*), a passerine with plumage polychromatism maintained by disassortative mating. The two distinct color morphs differ with respect to reproductive strategy in that white-striped birds invest more in territorial aggression than tan-striped birds. Whether territorial aggression in this species is biased according to the morph of an intruder is less understood. We found that during peak territorial and mating activity, both color morphs and sexes can exhibit aggression bias, but whether they do so depends on the strategy (morph) of the intruder. During simulated territorial intrusions, resident white-striped males and tan-striped females, which represent the opposite ends of a continuum from high to low territorial aggression, altered their territorial responses according to intruder morph. Tan-striped males and white-striped females, which represent the middle of the continuum, did not show a bias. We propose that because of the disassortative mating system and morph differences in reproductive strategy, the fitness risks of intrusions vary according to the morphs of the resident and the intruder, and that aggression bias is an attuned response to varying threats to fitness.

## Introduction

**Figure 1:**
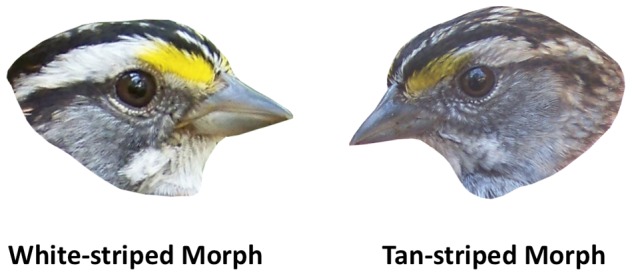
Plumage polymorphism in the white-throated sparrow. In both sexes, the white-striped morph (WS; left) has alternating black and white crown stripes, brighter yellow lores, and a clearer white throat patch. The tan-striped morph (TS; right) has alternating brown and tan crown stripes, duller lores, and dark bars within a duller throat patch. Photos by Christopher Gurguis.

Polymorphic species in which discrete genetic variants exhibit alternative phenotypes can provide valuable insight into the evolutionary causes and maintenance of the diversification of form and function [1]–[3]. In many vertebrate species, coexisting color morphs are characterized by differences in territoriality, aggression, mating effort, or parental care [2], [4]–[5]. One such example is the white-throated sparrow (*Zonotrichia albicollis*), a passerine bird in which genetic color morphs (Fig. 1) adopt alternative reproductive strategies. In both sexes, white-striped (WS) birds engage in more territorial defense but less parental care than tan-striped (TS) birds [6]–[9]. WS males invest relatively more in extra-pair mating whereas TS males invest more in mate guarding [10]–[11]. The two morphs thus represent two ends of a classic trade-off in life history strategies, with investment in territorial aggression and mating effort at one end and in parenting at the other [12].

When discrete color morphs differ in life history strategies, coloration may signal to others an individual's likely behavior in imminent encounters [2], [13]. The dynamics of social interactions in polymorphic species are thus expected to depend in part on the morph of the individuals involved. Most authors investigating social interactions in polymorphic species have focused on the role of color morph in mate choice (e.g., [14]) or on the outcome of agonistic interactions (e.g., asymmetric dominance; [13], [15]). Fewer have tested whether and how individuals of different morphs alter their aggressive responses in competitive encounters according to the morph of their opponent [16]–[18]. Such “aggression bias” has been hypothesized to stabilize phenotypic polymorphisms by contributing to negative frequency-dependent selection [16], [19]–[20]. In white-throated sparrows, however, color polymorphism is maintained not by frequency-dependent selection but by disassortative mating [21], in which the members of a breeding pair are nearly always of opposite morph. This system provides an opportunity to consider the evolution of aggression bias under a different selection regime.

Aggression can be costly [22], so the degree to which individuals engage in it should depend on the strategy of the intruder and the associated threat to the resident's reproductive fitness. According to this hypothesis, aggression associated with territorial defense in species with morph-specific reproductive strategies will depend on the morph of the intruder as well as the resident. We tested this prediction by comparing the responses of free-living white-throated sparrows to simulated territorial intrusions by males of the two color morphs during peak territorial and mating activity. If the magnitude of the response to an intrusion reflects the fitness risks of that intrusion, responses should vary according to the morphs of both the resident and the intruder.

## Methods

### Ethics Statement

The research methods described herein were approved by Emory University's Institutional Animal Care and Use Committee (protocol #DAR-2000739) and adhere to NIH standards and the Ornithological Council's Guidelines for the Use of Wild Birds in Research. Permits to conduct this field study on white-throated sparrows were issued by the Maine Department of Inland Fisheries and Wildlife (#2010-295 and #2011-295), the U.S. Geological Survey (#23369), and the U.S. Fish and Wildlife Service (#MB009702). Permission to conduct this research in the Hemlock Stream Forest was granted by the Forest Society of Maine.

### Data Collection and Analysis

We conducted simulated territorial intrusions (STIs) during May 2010 and 2011 in the Hemlock Stream Forest in Argyle, Maine, USA (45.092823° N, −68.689764° W). This sampling period followed territory establishment and pair formation, coincided with the pre-laying, nest-building, and egg-laying stages, and generally preceded incubation in the population. Thus, STIs were conducted when a large proportion of females were presumed fertile [23], and when mating effort and territorial responses to intrusion are greatest in this species ([10]; D. Loncke unpubl. data, as cited in [24]). Pair types were either WS male X TS female (n = 14) or TS male X WS female (n = 17); no same-morph pairs were observed during this study.

Several days prior to an STI, we captured and color-banded the focal territorial male (and female when possible) for identification during the STI. We performed STIs by placing a live, caged decoy centrally in a known territory and broadcasting conspecific song (playback) from an LG® portable stereo speaker placed next to the cage and controlled remotely with an Apple Ipod Nano®. In this species, a decoy accompanied by playback elicits a stronger territorial response than does a decoy or playback alone [25]. To avoid pseudoreplication [26], we used 21 male decoys (12 TS, 9 WS) and 12 different song exemplars that were unfamiliar to the residents. Decoys spent most of their time during STIs feeding and preening; they did not display any of the aggressive behaviors or vocalizations exhibited by the residents (described below). Song exemplars from singers of unknown morph were downloaded from the Borror Laboratory of Bioacoustics and edited according to Maney et al. [3] to equalize volume and eliminate background noise. In this species, individual variation in song can be attributed primarily to the use of ascending *versus* descending consecutive syllables and to pitch; no variation in these or other parameters can be attributed to morph [24]. Local dialects are not observed [27], but pitch varies according to habitat type [28]–[29]. To control for possible unknown effects of song structure in this study, we used exemplars that varied according to recording locale, pattern of ascending *versus* descending syllables, and pitch. Each decoy was randomly assigned one song exemplar, and that song was used for only one decoy each year. Playback during STIs consisted of the one song repeated every 15 s.

We performed two STIs on each territorial pair on consecutive days between 06:00–011:30 EDT. We presented a TS male decoy for one STI and a WS male decoy for the other such that the order of presentation was counterbalanced. We conducted the two STIs at the same time of day for a given pair, but balanced time of day across pairs so that both pair types received earlier and later STIs. We did not conduct STIs on immediate neighbors on the same day. Each STI was monitored by two observers positioned 30 m apart and on opposite sides of the decoy.

Once playback commenced, we conducted the STI for 10 min after detecting the territorial male within 30 m of the decoy. For resident birds, we recorded five behaviors that are commonly used to define territorial aggression in this and other *Zonotrichia* species [7], [30], [31]. In response to STI, more aggressive residents approach the decoy sooner, get closer, fly over more often, and spend more time in proximity [7], [30], [31]. We therefore scored latency to approach (i.e., time from playback start until the resident approached within 15 m of the decoy), number of flights directly over the decoy, time spent within 5 m from it, time spent within 2 m, and closest approach distance. In addition, we scored three vocalizations that are known to signal aggression in this species [24]: song, which is the primary vocalization used to repel intruders in this and related sparrow species [32], [33], chip (or pink) calls, which are alarm calls used frequently during agonistic encounters [24], and trills, which also occur during agonistic encounters, particularly in response to an intruder's song [24].

Both male and female white-throated sparrows exhibit all of these aggressive behaviors in response to STI [7], [24], [25], [34], so we scored all of the behaviors for both members of each pair. Females may also perform a copulation solicitation display (described in [24]), but only one female in this study solicited a male decoy. Thus, the females' responses were also largely aggressive in nature.

A single behavior alone does not define territorial aggression; thus, we used principal components analyses (PCAs; Table 1) to construct a composite physical aggression score (PC1) from the five physical behaviors and a composite vocal aggression score (PC1) from the three vocalizations (*sensu* [30], [35]). Although males and females in this species exhibit the same aggressive behaviors, levels of aggression are sex-dependent [24]. Thus, we conducted PCAs and subsequent statistical analyses separately for each sex. We analyzed aggression scores (PC1s) with mixed-model ANOVAs; fixed effects were resident morph (TS or WS), intruder morph (same or opposite), and a resident*intruder interaction. Year was included as a random effect, and resident ID was included as a nested random effect to control for repeated sampling. When significant effects were found, we used orthogonal contrasts within morph-sex type to compare the aggressive responses (LS means of aggression scores) to same-morph intruders with those to opposite-morph intruders. We also examined whether specific behaviors (e.g., song rate) depended on intruder morph. Behavioral data were not normally distributed, so we used Wilcoxon Signed-Rank tests (WSR) within morph-sex type to evaluate the effect of intruder morph on each behavior. Statistical analyses were performed using JMP v. 8.

**Table 1:** Principal component factor loadings for the analysis of (a) vocal and (b) physical aggressive behaviors of male and female white-throated sparrows to simulated territorial intrusions.

*(a)*	Vocal Response	Male PC1	Female PC1
	Songs	−0.71	−0.16
	Chip calls	0.58	0.72
	Trills	0.40	0.67
	*Variance Explained*	*44%*	*39%*

*(b)*	Physical Response	Male PC1	Female PC1
	Latency to approach	−0.30	−0.41
	Flights over decoy	0.45	0.38
	Time within 5m	0.51	0.50
	Time within 2m	0.46	0.44
	Closest approach	−0.49	−0.49
	*Variance Explained*	*53%*	*65%*

The percentage of variation in these responses explained by the first principal component (PC1) is noted in italics.

## Results

**Figure 2:**
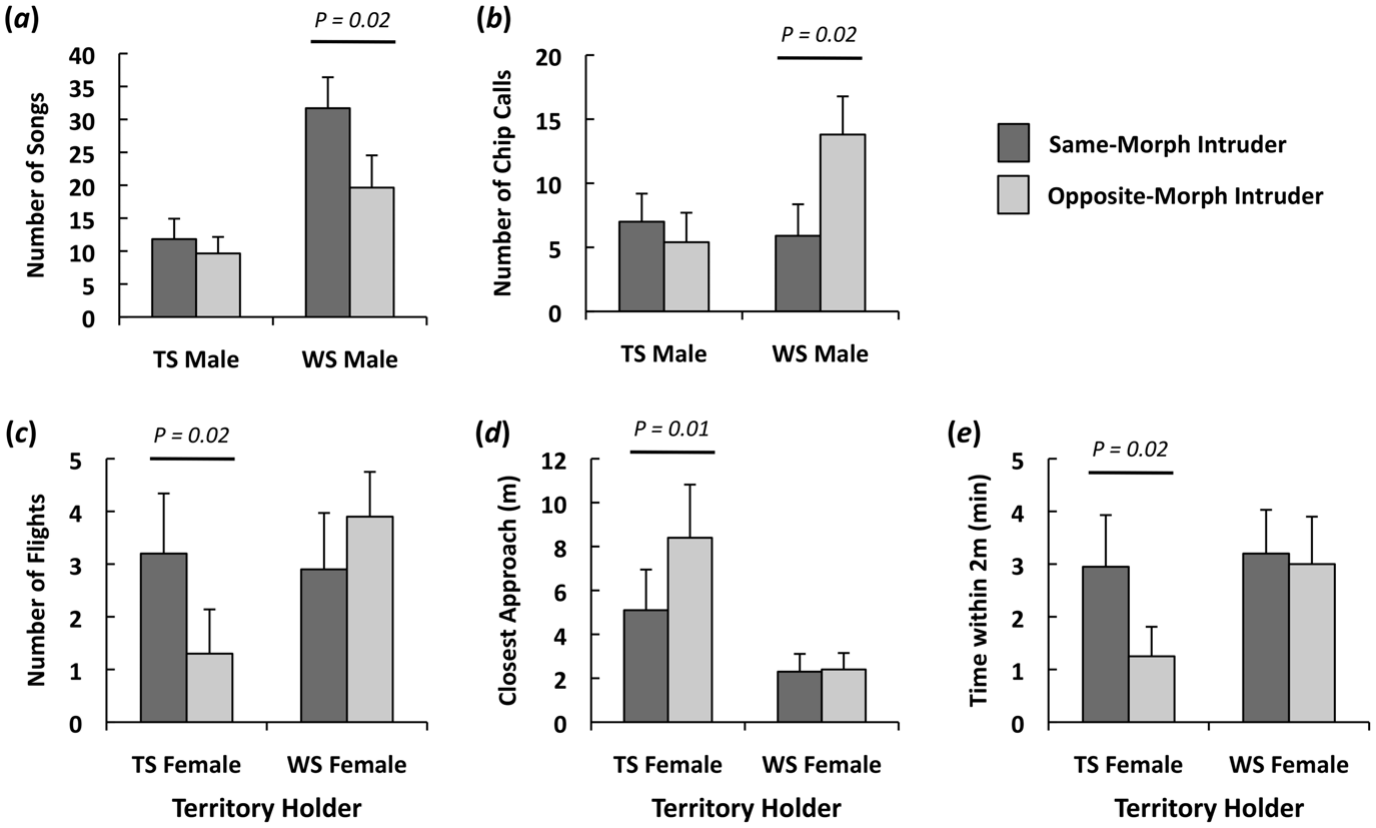
Variation in the behavioral responses of territorial white-throated sparrows to same-morph and opposite-morph male intruders. (a–b) Vocal behaviors of resident tan-striped (TS; n = 17) and white-striped (WS; n = 14) males. (c–e) Physical behaviors of resident TS (n = 14) and WS (n = 17) females. Values are means ± SE, and *P*-values are from WSR tests used to compare responses to same-morph intruders with those toward opposite-morph intruders.

In the analysis of male physical aggression, PC1 explained 53% of the variation, and in the analysis of male vocal aggression, PC1 explained 44% of the variation (Table 1). Intruder morph did not affect the physical aggression of resident males (intruder main effect: F_1, 29_ = 0, p = 0.98; resident main effect: F_1, 28_ = 0.3, p = 0.57; resident*intruder interaction: F_1, 29_ = 0.7, p = 0.42; Table 2). In contrast, resident males altered their vocal aggression according to intruder morph depending on their own morph (intruder main effect: F_1, 29_ = 7.0, p = 0.01; resident main effect: F_1, 28_ = 0.9, p = 0.35; resident*intruder interaction: F_1, 29_ = 5.6, p = 0.03). Overall, WS males mounted stronger vocal responses towards same-morph intruders than towards opposite-morph intruders, whereas TS males displayed similar vocal responses to both intruder morphs (Table 2). When specific vocalizations were considered, WS males sang more in response to same-morph intruders (WSR, Z = 37.5, p = 0.02; Fig. 2a) but gave more chip calls in response to opposite-morph intruders (WSR, Z = 22.5, p = 0.02; Fig. 2b).

**Table 2:** Composite physical and vocal aggression scores for resident white-throated sparrows during simulated territorial intrusions by males of the two color morphs.

	Vocal Aggression Score (PC1)			Physical Aggression Score (PC1)		
Resident	Same-morph	Opposite-morph	*p* value	Same-morph	Opposite-morph	*p* value
	Intruder	Intruder		Intruder	Intruder	
TS Male	0.11±0.01	0.17±0.20	0.83	0.01±0.36	−0.26±0.57	0.55
WS Male	**−0.67±0.36**	**0.34±0.36**	**<0.01**	0.01±0.31	0.30±0.32	0.57
TS Female	−0.01±0.31	−0.07±0.25	0.86	**−0.01±0.59**	**−0.87±0.73**	**0.02**
WS Female	0.20±0.33	−0.13±0.25	0.33	0.84±0.34	0.93±0.31	0.65

Aggression scores are PC1 data (means ± SE) generated from principal components analyses (see Table 1). *P*-values are from orthogonal contrasts following mixed-model ANOVAs.

For females, PC1 explained 65% of the variation in physical aggression and PC1 explained 39% of the variation in vocal aggression (Table 1). Whether resident females altered their physical aggression according to intruder morph depended on their own morph (intruder main effect: F_1, 21_ = 2.6, p = 0.12; resident main effect: F_1, 23_ = 2.9, p = 0.10; resident*intruder interaction: F_1, 21_ = 4.7, p = 0.04). TS females exhibited a stronger physical response to same-morph intruders than to opposite-morph intruders, whereas WS females did not show a bias (Table 2). Across specific physical behaviors, TS females exhibited more flights (WSR, Z = 17.0, p = 0.02; Fig. 2c), a closer approach (WSR, Z = 24.5, p = 0.01; Fig. 2d), and spent more time within 2 m of the decoy (WSR, Z = 14.0, p = 0.02; Fig. 2e) when presented with a same-morph intruder. In contrast, intruder morph had no statistical effect on the vocal aggression of resident females (intruder main effect: F_1, 27_ = 0.7, p = 0.42; resident main effect: F_1, 26_ = 0.1, p = 0.93; resident*intruder interaction: F_1, 27_ = 0.3, p = 0.58; Table 2).

## Discussion

Our results showed that white-throated sparrows of both sexes and morphs can adjust their aggressive behaviors according to the morph of their opponent. In WS males and TS females, this aggression bias depended on the morph of the resident in that aggressive responses were stronger towards same-morph intruders. Because intruders that compete for food or nest sites should affect both morphs equally, our finding of morph-dependent aggression bias may be driven by other factors such as the disassortative mating system. Same-morph pairs are exceedingly rare [24], and none were observed during this study. Thus, for a resident female, an opposite-morph male intruder may represent a better mating opportunity, thereby warranting a less aggressive response than a same-morph male. That less physical aggression was exhibited by TS females toward WS than TS intruders (Fig. 2c–e) supports this hypothesis. For the resident male, a same-morph intruder would be stronger mate competition, and WS males, which show higher aggression toward WS than TS intruders, may be responding to this threat. Alternatively, aggression bias that depends on the morph of the resident could reflect morph-typic strategies for dealing with potentially hostile intruders. Perhaps WS males escalate their own aggression when challenged by high aggression WS intruders, whereas TS females reduce their aggression towards WS intruders as an avoidance strategy. Since the captive decoys in this study did not exhibit threatening behaviors, residents were not likely responding to genuine threats, but instead to perceived threats signaled by the decoy's color.

Although WS males and TS females exhibited aggression bias, TS males and WS females did not, suggesting that factors other than the disassortative mating system and intruder aggression are also important. Among morph-sex types in this species, WS males are the most aggressive and TS females are the least; TS males and WS females exhibit intermediate levels of aggression [6]–[9]. We observed aggression bias only at either end, but not in the middle, of this continuum. Aggression could be most costly at these extremes, and the behavioral biases reported here may act to reduce aggression-related costs in WS males and TS females. The lack of aggression bias in TS males and WS females could also be driven by morph differences in mate-seeking strategies. TS males should be aggressive towards TS male intruders, since they are the preferred male of their WS female mates [36]. Although WS intruders should spark less interest from WS females than TS intruders [36]–[37], their high extra-pair mating effort [10], [11] may nonetheless warrant aggressive responses from TS resident males. TS males in this study were equally aggressive to intruders of both morphs, which likely reflects an intense and unbiased mate guarding effort. Similarly, female mate-seeking strategy may explain the lack of aggression bias in WS females. WS females are less likely to accept EPCs than are TS females, as evidenced by their higher aggression towards male intruders [7] and fewer extra-pair young in their nests [10]–[11]. Reduced interest in EPCs may result in unbiased aggressive responses towards intruders regardless of morph (Fig. 2c–e).

In a previous intrusion study [7], conducted during the nestling phase when the risk of cuckoldry is largely absent, parental WS females were more likely to attack WS than TS models. Our contrasting findings suggest that the expression of aggression bias may differ across breeding stages. Mate-seeking strategy is less relevant during the nestling stage, and responses to intruders may be more easily influenced by the threat of intruder aggression. Overall, available evidence suggests that aggression bias depends on the morph, sex, and reproductive stage of the resident and may moderate social dynamics throughout reproduction. Outside the breeding season, aggression does not appear to be adjusted according to the morph of the opponent [38].

Our findings support the hypothesis that the degree to which white-throated sparrows aggressively defend against intruders depends on both the strategy of the intruder and the associated threat to the resident's reproductive fitness. Mounting evidence suggests that morph-based aggression bias is an important evolutionary force in phenotypic diversification and speciation, particularly in systems evolving under negative frequency-dependent selection (reviewed in [20]). Our current results demonstrate that aggression bias likely serves an important function under other selection regimes as well. Because morph-biased aggression may require individuals to recognize self-morph as well as their opponent's morph, future research should test the cognitive mechanisms underlying the perception of self versus opponent morph [39].
